# Treatment of Pulpless Teeth

**Published:** 1889-02

**Authors:** Frank Hampton Goffe


AMERICAN JOURNAL

—OF—

DENTAL SCIENCE.

VOL. XXII. THIRD SERIES—FEBRUARY, 1889. NO. 10.

ARTICLE I.

### TREATMENT OF PULPLESS TEETH.

BY FRANK HAMPTON GOFFE, L. D. S. ENG. and EDIN.

[A paper read before the Birmingham Dental Students' Society.]

As it is the ill fortune of many to have teeth come under their care with either exposed or dying pulps, I thought that it might be interesting to put before you the various methods that have been recommended for their treatment.

The arguments for and against capping still go on, but it seems to me that there is a growing tendency to destroy all pulps exposed by caries—for a long time past I have given up trying to save exposed pulps.

Capping is very apt to fail from the fact that septic fluids have already been in contact with the pulp, and you cannot determine the date of exposure and injury; so when the pulp has been capped some time you find it becomes sensible to hot and cold fluids, then periostitis ensues. Our knowledge of the tooth structure has made us not so anxious to preserve the pulps, as we know that the dentine is in active communication with the cementum, which again derives its nutriment from the pericemental membrane, so that the tooth is not dependent upon the pulp for its life; but still we must remember that there is more work thrown on the vessels of the lining membrane after the death of the pulp.

Your first attention to a carious tooth is to remove the *debris* trom the cavity, then syringe it out with a weak carbolic solution. Now apply the rubber dam, which is one of the most important factors in antiseptic surgery for filling teeth, for by it the tooth is cut off from many sources of infection, and the drug used is not weakened or neutralized by the saliva.

Now, having the rubber fixed in position, you can remove the decay, and if the pulp is exposed, my practice is to wound it to induce hæmorrhage, treating it with carbolic acid to remove any coagulum, in order to allow your dressing to be in absolute contact with the pulp. In the case of the front teeth you can apply cocaine dissolved in carbolic acid for two or three minutes, when the nerve can be removed with very little pain.

In order to effect devitalization the usual dressing is Baldock's nerve paste. The following recipe is the one I generally use:—

R Acidi Arseniosi gr. xii.
Morphiæ Acetatis gr. ij.
Creasoti q. s.
Misce.

I find that the intense pain is usually allayed in about a quarter of an hour. I always leave the dressing in for two or three days; it is a great fallacy for dentists to insist on seeing their patient the following day, as the small quantity of arsenic used cannot possibly do any harm if it is sealed up securely; and it seldom renders a second application necessary. Occasionally, however, a small portion of undevitalized nerve still remains at the extremity of the canal, which the application of cocaine and carbolic acid will enable you to remove by means of a nerve extractor annealed in the spirit flame and passed to the apex of the fang; any remaining tissue should be removed with a Donaldson's nerve canal cleaner, subsequently drying with a hot air syringe.

The canal should then be enlarged with flexible drills or reamers, carefully avoiding forcing any *debris* through the foramen.

It need hardly be said that all the instruments should be quite clean and rendered aseptic; this can be done by wiping them with a solution of carbolic acid—1 in 40.

After cleaning the fangs, hot air should be injected for some time to destroy any lurking mico-organisms. A question now arises whether you should dress the fang before finally filling, which if you determine upon, one or other of the subjoined antiseptic drugs should be applied, viz., the essential oils eucalyptus or cloves, together with iodoform or the time-honoured carbolic acid.

The most powerful antiseptic agent we have is bichloride of mercury. A very good way of using it is a solution in alcohol 2 grs. to the ounce. According to Dr. Miller's table the antiseptic drugs act as follows :—

| | Development of fungi arrested. |
|---|---|
| Bichloride of mercury - - | 1 in 100,000 |
| Peroxide of hydrogen - - | 1 in 8,000 |
| Iodoform - - - - | 1 in 5,000 |
| Eucalyptus - - - - | 1 in 600 |
| Carbolic - - - - | 1 in 500 |
| Arsenic - - - - | 1 in 250 |

Here I might mention the new drug iodol. All the therapeutical properties of iodoform are claimed for it, but without its disagreeable smell or taste; it is a light fawn-coloured substance, which is almost insoluble in water, but freely so in ether or chloroform and olive oil; it contains about 90 per cent. of iodine.

Iodol can also be used as iodolized wax, which is a very good substitute for those who use iodoform and wax for fang filling, as it melts at a lower temperature and is more easily packed.

The system advocated by Mr. Coleman is to thoroughly clean out the pulp chamber; then apply a minute quantity of arsenic and proceed to fill the cavity in the ordinary way. It seems to answer well with some practitioners, as arsenic is a powerful antiseptic.

Another method is to mix together oxychloride of zinc and iodoform, then fill the fangs, using strands of wool to thicken it; this is apt to cause some amount of irritation for a short time, so you can substitute the oxyphosphate for the oxychloride of zinc. A solution of gutta percha and iodoform in chloroform, or the root fillings of gutta percha made by the S. S. White Co. are also useful as root fillings. I think one of the easiest to use is plaster of Paris mixed with iodoform and water, and worked into the canals with a few strands of wool; it seems to answer the purpose perfectly.

A ready but less perfect way of treating pulpless teeth is by the operation of rhizodontrophy; that is, after cleaning out the pulp cavity and fangs to cap over them, and then with a sharp instrument drill a perforation level in the gum into the fangs, thus leaving a drainage tube for the escape of gas or fluids.

If the foregoing plans are followed carefully, rendering all instruments, &c., aseptic before using, I think there is very little fear of any complications arising afterwards. As inflammation is most apt to occur during the first few weeks after filling, a few capsicum plasters should be given to the patient, with instructions to apply them in case of any irritation arising.

Teeth occasionally come under our charge with pulps already dead, without inflammatory effusion of the gum, but with pulps dead and an abscess formed or forming.

First thoroughly open up the pulp chamber, in order to gain direct access to the canals; loose *debris* should be washed out with weak carbolic solution, and the canals thoroughly cleaned with canal cleaners and a Gates Gliddon drill, which has the great advantage that it is a blunted drill and not likely to go through the apical foramen.

Peroxide of hydrogen should be pumped up to the end of the roots by means of a broach with a little twisted cotton wool round it until all odour has entirely disappeared.

Bichloride of mercury 1 in 1,000 can be used in the same way, alternately with the peroxide of hydrogen, or a mixture of the two may be used thus:—

Hydrogen peroxide f. ℥.
Mercury perchloride gr. ij.

In treating these cases it is very important to use the hot air syringe after every application of the hydroxyl.

The peroxide of hydrogen is a very unstable compound and so should be quite fresh and kept away from the sunlight, which decomposes it. It very readily gives off oxygen, being itself converted into water; this oxidizes or destroys all the micro-organisms and pus cells. Therefore it is advisable to continue pumping it up as long as the frothy bubbles appear; when these cease you can wipe the fangs out with chloroform and proceed to fill the roots with whatever materials you prefer. Peroxide of hydrogen is not a new antiseptic; it was introduced as a medicine by Dr. Richardson in 1858, and as an antiseptic for dental surgery by Dr. Coffin in 1883.

There is no question that the immediate treatment is gaining; and when the pulp has been recently destroyed with arsenic, and in cases of patients coming some distance, when a visit more or less is of importance, this treatment is invaluable.

If an abscess has already formed, the apical foramen should be enlarged and hydroxyl and perchloride of mercury pumped through the foramen until there is no discharge of pus down the root and no froth appears; this will usually occupy about ten minutes. If the canals are too small to admit the broach, it is far better to enlarge them till you can pass your instrument, as you must have clear openings to effect a perfect cure.

Immediately the roots are quite clean and aseptic, I proceed to fill them, as I think very little is gained by dressing the root time after time. As soon as the canals are rendered aseptic, the cause of the abscess is taken away, and with proper treatment, the cause being removed, the effect can also be quickly remedied.

The best and quickest way, if all the pus cannot be brought away through the root, is to open the abscess and remove the diseased part through the alveolus.

Formerly, this caused considerable pain, but now it can be performed quite painlessly. I take 1 gr. of hydrochlorate of cocaine and dissolve it in carbolic acid, and inject it with the hypodermic syringe. Dr. Vian pointed out that carbolic was itself a good local anæsthetic, and with this solution I have had very good results.

After waiting three to five minutes the alveolus should be perforated and an opening made into the abscess; this cavity should be syringed out with peroxide of hydrogen, and if the abscess is very large or chronic, I plug the sinus with lint soaked in carbolic.

At times great benefit will be derived from a little medical treatment, the sulphides having a great effect on suppuration; the best of these is the calcium sulphide, which will often give the patient considerable relief.

I have put before you, as well as I am able in such a short paper, the various methods for the preservation of teeth after the death of the pulp; the retention of the roots in a healthy state will become no doubt of greater consequence as they are utilized more and more for mounting crowns and bridge-work.—*The Dental Record.*

